# Light and the Brain: A Clinical Case Depicting the Effects of Light on Brainwaves and Possible Presence of Plasma-like Brain Energy

**DOI:** 10.3390/brainsci14040308

**Published:** 2024-03-25

**Authors:** Zamzuri Idris, Zaitun Zakaria, Ang Song Yee, Diana Noma Fitzrol, Muhammad Ihfaz Ismail, Abdul Rahman Izaini Ghani, Jafri Malin Abdullah, Mohd Hasyizan Hassan, Nursakinah Suardi

**Affiliations:** 1 Department of Neurosciences, School of Medical Sciences, Universiti Sains Malaysia, Kubang Kerian 16150, Kelantan, Malaysia; zaitunzakaria@usm.my (Z.Z.); happy_4428@hotmail.com (A.S.Y.); diana_noma@hotmail.com (D.N.F.); ihfazrose5919@usm.my (M.I.I.); yoppghani@gmail.com (A.R.I.G.); brainsciences@gmail.com (J.M.A.); 2 Brain and Behavior Cluster (BBC), School of Medical Sciences, Universiti Sains Malaysia, Kubang Kerian 16150, Kelantan, Malaysia; 3 Hospital Universiti Sains Malaysia (HUSM), Universiti Sains Malaysia, Kubang Kerian 16150, Kelantan, Malaysia; hasyizan@usm.my; 4 Department of Anesthesiology, School of Medical Sciences, Universiti Sains Malaysia, Kubang Kerian 16150, Kelantan, Malaysia; 5 School of Physics, Universiti Sains Malaysia, Gelugor 11800, Penang, Malaysia; nsakinahsuardi@usm.my

**Keywords:** light, photobiomodulation, brainwaves, thermodynamics, quantum brain, plasma, black body, awake brain surgery, electrocorticography

## Abstract

Light is an electromagnetic radiation that has visible and invisible wavelength spectrums. Visible light can only be detected by the eyes through the optic pathways. With the presence of the scalp, cranium, and meninges, the brain is seen as being protected from direct exposure to light. For that reason, the brain can be viewed as a black body lying inside a black box. In physics, a black body tends to be in thermal equilibrium with its environment and can tightly regulate its temperature via thermodynamic principles. Therefore, a healthy brain inside a black box should not be exposed to light. On the contrary, photobiomodulation, a form of light therapy for the brain, has been shown to have beneficial effects on some neurological conditions. The proposed underlying mechanisms are multiple. Herein, we present our intraoperative findings of rapid electrocorticographic brainwave changes when the brain was shone directly with different wavelengths of light during awake brain surgery. Our findings provide literature evidence for light’s ability to influence human brain energy and function. Our proposed mechanism for these rapid changes is the presence of plasma-like energy inside the brain, which causes fast brain activities that are akin to lightning strikes.

## 1. Introduction

**Figure 1:**
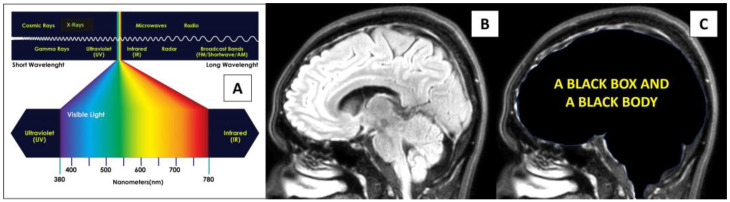
Light and the brain. (**A**) The electromagnetic spectrum of light. (**B**,**C**) A healthy brain receives light through the eyes and lies inside a black box, thus behaving like a black body with thermodynamic equilibrium and having properties that can tightly regulate its temperature.

Light is the opposite of darkness, and it is meaningful to followers of many religions across the world such as the festival of light (Diwali) for Hindus, the candle festival for Buddhists, and as a symbol of God and the first creation (metaphorical light or Nur in Arabic) for the Abrahamic religions. In the academic arena, light has been viewed as an electromagnetic force or energy or waves since the great Scottish mathematical physicist James Clerk Maxwell proved for the first time in history the unification of electric (with field) and magnetic (with vortex) forces. Scientists view electromagnetic light with its highest speed at approximately 300,000 km/s as fundamental to our health and life [1,2,3,4,5]. Our most important source of natural light is the sun. The sunlight or white light is in fact composed of visible and invisible light frequency spectrums. Visible light (which can be processed by our visual system) has wavelengths from about 380 nanometers (violet) to about 780 nanometers (near-infrared). In terms of frequency, this corresponds to a band in the vicinity of 400 (near-infrared) to 790 (violet) terahertz. This visible band has seven main spectral colors including red, orange, yellow, green, cyan, blue, and violet. With reference to the brain, light can only pass through the eyes to enter the brain. Layers of scalp, cranial bone, and meninges form a protective barrier from light. The light-protective cranial layers are therefore seen as necessary to protect the brain from harmful effects of direct light exposure. Cakir et al. performed an animal study with the aim of evaluating the effects of various experimental lights on the viability of cultured neurons. They found that low-dose gamma rays and long-term visible light induce remarkable neuronal apoptosis and cell death, but no significant effects were observed with laser light [6]. In addition to the biological perspective of light interaction with one of the brain’s anatomical components (neurons), the physical perspective of light interaction with the brain’s physiological component or electromagnetic field (brainwaves) is also worth noting. A recent physics study by Maccaferri et al. in 2020, demonstrated nanoplasmon formation when light interacts with electrons [7]. With these unfavorable biological and interesting physical effects, a black box created by the cranium seems suited to house the brain (Figure 1). In sum, it seems like the healthy brain should not be exposed directly to external light. Herein, we present some interesting brainwave findings during awake cranial surgery when the brain was shone directly with various wavelengths of light. Our findings may provide strong evidence for further studying the details of using light as a mode of therapy for neurological disorders in humans and may invigorate scientific discussions on brain thermodynamics and plasma-like brain energy.

## 2. Case Report

### 2.1. Clinical Description

**Figure 2:**
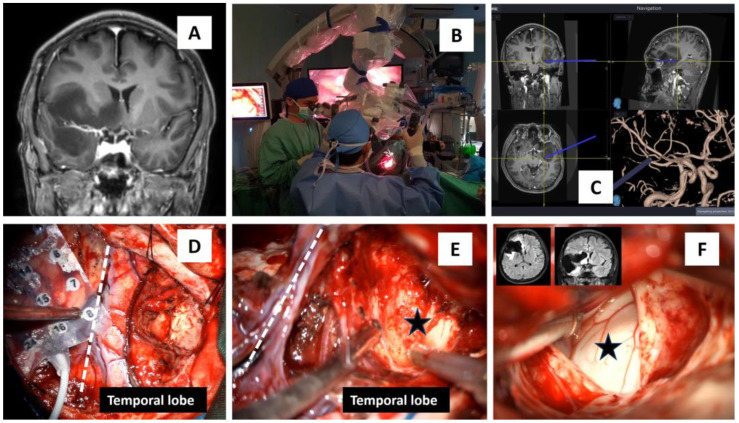
Awake operative procedure for a right insular glioma surgery. (**A**) MRI T1-coronal view depicting the right insular glioma that spread to the ipsilateral basal ganglia, midline basal forebrain nuclei, and temporal lobe. (**B**) Use of a microscope with xenon white, blue, and near-infrared coherent laser light for autofocus. (**C**) A modern neuronavigation system with coronal, sagittal, axial, and vascular images was utilized in removing the tumor. (**D**–**F**) An 8 × 4 grid with 32 electrodes was placed over the right frontal lobe, and the surgery was first started at the temporal lobe, which gradually exposed the hippocampus (black star in (**E**,**F**)). The two MRI images in (**F**) are the post-surgery images that showed near-total debulking of the tumor, and the white dashed line in (**D**,**E**) marks a Sylvian area that divides the frontal from the temporal lobe.

A 32-year-old unmarried man was referred to our hospital for awake right insular glioma surgery. He initially presented with intermittent headaches for the last 2 years with infrequent episodes of generalized motor seizures. His physical examination did not disclose significant neurological deficits; however, neuropsychological assessment (Comprehensive Trail-Making Test) revealed significant impairment in attention and concentration. Neuroimaging revealed an 8.7 × 6.0 × 5.4 cm right-sided intra-axial mass involving the right frontal, temporal, insular, and part of the ipsilateral caudate head. An opened biopsy performed in another center prior to referral revealed a diffuse grade II astrocytoma. Before surgery, a detailed explanation was given to the patient and informed consent was obtained for awake brain surgery, intraoperative brainwave monitoring, the use of a computerized neuronavigation system, and microsurgery using a standard neurosurgical microscope (Zeiss OPMI Pentero) equipped with two laser dots light with size of 2–3 mm each, white light, and blue light to remove the tumor. During surgery, the neurosurgeon used an 8 × 4 electrocorticographic (ECoG) grid electrode, which was placed directly onto the ipsilateral frontal lobe to detect early seizure brainwaves and to assess the state of consciousness of the patient. Surgery was initially started at the right temporal lobe with the removal of the tumor until the right hippocampus was exposed (Figure 2). No fluorescent agents were used for this surgery. The surgery was uneventful and near-total debulking of the tumor was achieved (Figure 2F). Histopathological diagnosis confirmed an isocitrate dehydrogenase (IDH) mutant astrocytoma with gemistocytic differentiation. Regarding prognosis, the average survival period for this tumor is shorter than an IDH mutant astrocytoma without gemistocytic differentiation (2 years versus 6 years, respectively) [8].

### 2.2. Awake Brain Surgery—Marked and Rapid Brainwave Changes When the Brain Was Directly Exposed to Different Wavelengths of Light

**Figure 3:**
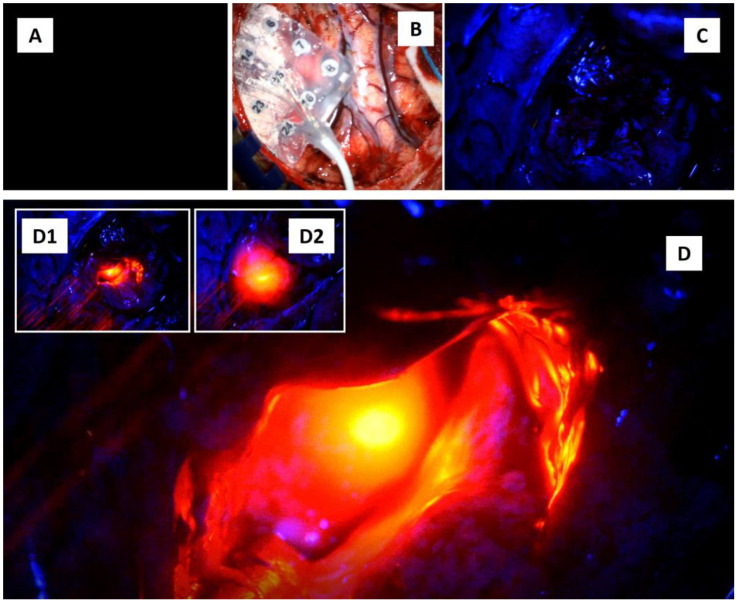
Shining the brain with four different lights. (**A**) The light is off. (**B**) The white light is on. (**C**) The blue light is on. (**D**) The blue light was superimposed with near-infrared autofocus laser light. (**D1**,**D2**) show additional intraoperative images in which the infrared laser light was shone onto the right hippocampus.

**Figure 4:**
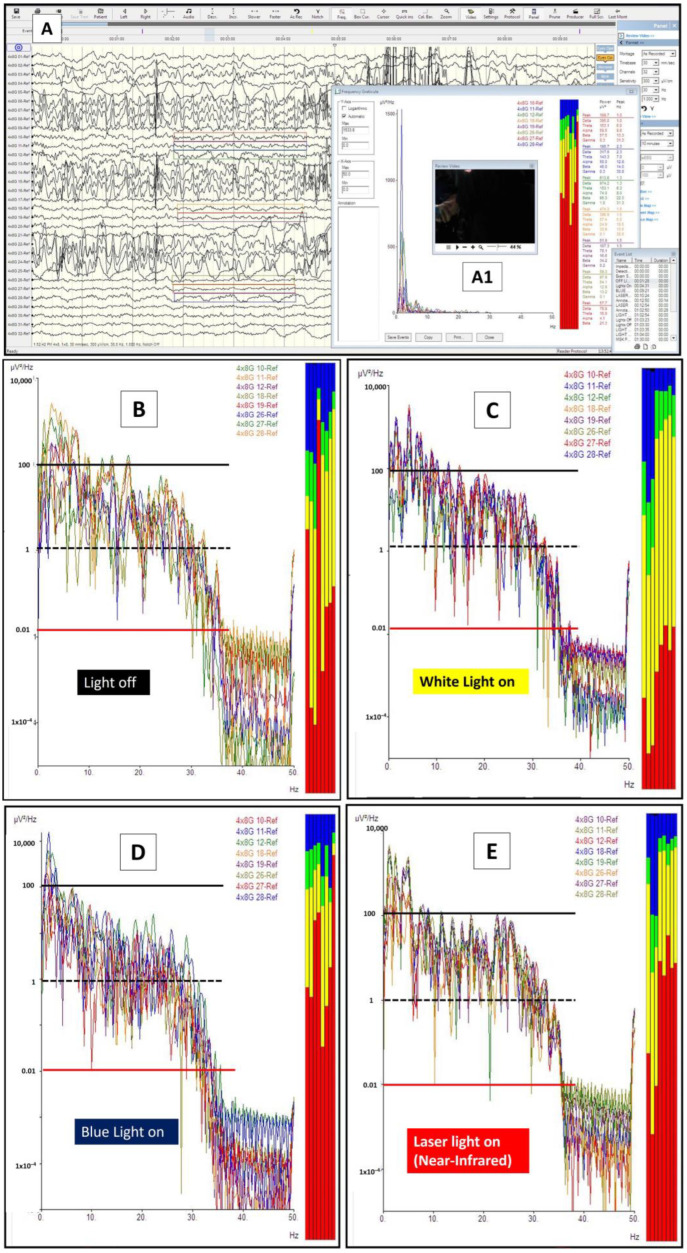
Light and brainwaves. (**A**) The brainwaves obtained via 8 × 4 grid electrodes during the procedure. Electrodes 10–12, 18, 19, and 26–28 were selected for brainwave log-power analysis. The off-light (**A1**) brainwaves were marked on the screen and were analyzed later for the log-power morphology and frequency pattern (**B**). A similar analysis was also completed for xenon white light (on-light) (**C**), blue light (**D**), and blue light with superimposed near-infrared light (**E**). For the graphs in (**B**) to (**E**), the x-axis represents the brainwave frequency (0 to a maximum of 35 Hz), whilst the y-axis represents the log power of the waves (log values for the amplitude in voltage with a power of 2 divided with frequency or uV^2^/Hz). Thus, the represented colored lines on y-axis denote the following: a red line as 0.01; a black-dashed line as 1, and a black line as 100 uV^2^/Hz. For the brainwave spectra (colored boxes): red represents delta waves [0.5–4 Hz], yellow represents theta waves [4–8 Hz], green represents alpha waves [8–12 Hz], and blue represents beta waves [12–30 Hz].

At one point during the surgery, the neurosurgeon stopped the tumor debulking and changed the microscope lighting from being switched off (the whole operative room was also in darkness with obvious parameters readings only noted on anesthetic machines) to switching on the white (300 Watts xenon light at 30% intensity), blue (at 400 nm wavelength), and finally the near-infrared laser lights (at 780 nm wavelength for microscope autofocusing). The final part consisted of superimposing the laser red light hitting the exposed hippocampus with a background of blue light. The purposes were to assess any peculiar features of the tumor area when viewed under these different lights and to note any peculiar features of brainwaves when the brain was shone with these lights. Each phase lasted for three to four minutes and was marked on the ECoG screen machine (Figure 3 and Figure 4A,A1). These were performed in a successive manner without brain or grid disturbance. The ECoG brainwave analysis was then completed using the NicoletOne^TM^ system (Natus, Orlando, FL, USA) with adjustable sensitivity format ranging from 10 to 5000 µV/cm, low and high cuts of 1 and 50 Hz, and time-based of 30 mm/s. The brainwave power spectrum (or energy per unit of time) (µV^2^/Hz) was automatically calculated by the quantitative ECoG power band software available in the system.

The brainwave morphological features under these four brain conditions are depicted in Figure 4B–E. In referring to the figures, one should notice the presence of gross morphological differences in terms of brainwave log power when the brain is exposed to different lights (pay particular attention to the y-axis power lines of 0.01—red line; 1—black-dashed line, and 100—black line uV^2^/Hz when comparing them). Firstly, the brainwave log-power had a different morphology in the presence of light compared with those in darkness and a black box (B vs. C) (note: the brain in a cranium is an analogy to the brain in a black box). Secondly, the brainwave log-power morphology for the brain when directly exposed to blue light (400 nm wavelength) (D) and near-infrared light (780 nm wavelength, i.e., blue superimposed with laser near-infrared light) (E) were different compared with when it was in a black box (B). Finally, the brainwaves of the brain that were exposed to blue light had a contradictory feature when compared with near-infrared light exposure (D vs. E). The brainwave log power shifted to lower log power when exposed to blue light (with its mean power around 1 uV/Hz or a black-dashed line). On the other hand, the brainwave log power shifted towards higher log power when the brain was exposed to near-infrared light (with its mean log power lying between 1 and 100 uV/Hz or in between a black-dashed line and a black line). These differences were obviously noted when comparing them to the off-light brainwave log-power morphology (D and E vs. B).

**Figure 5:**
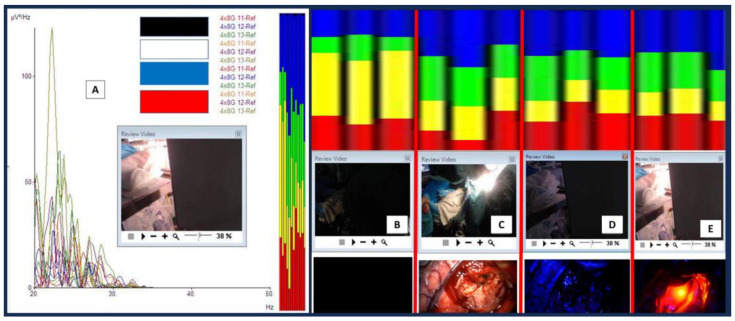
Brainwave frequency analysis. (**A**) A consecutive series of different light exposures on the exposed brain with brainwaves recorded at electrodes 11 to 13. (**B**–**E**) Four different columns represent brainwave spectra and different light exposures directly on the brain ((**B**) for when the light was off, (**C**) for white light, (**D**) for blue light, and (**E**) for near-infrared light). Take note that the laser infrared was directed towards the exposed hippocampus. For the brainwave spectra: red represents delta waves [0.5–4 Hz], yellow represents theta waves [4–8 Hz], green represents alpha waves [8–12 Hz], and blue represents beta waves [12–30 Hz].

**Figure 6:**
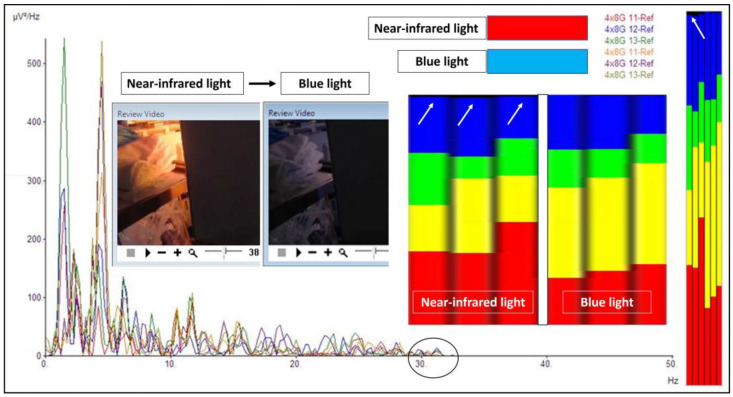
The difference between near-infrared and blue light. When near-infrared light was directed towards the hippocampus, gamma waves were noted in the frequency analysis. The gamma waves (a black circle on the graph with a range just above 30 Hz and black spectrum/white arrows in the frequency analysis) disappeared when the near-infrared light was off and shifted to the blue light. Note that a black-colored brainwave spectrum represents gamma waves (30–50 Hz in our analysis).

Further detailed analyses were completed for the obtained brainwaves. The frequency graph analysis is represented in Figure 5A as brainwave frequency (x-axis) versus brainwave power in uV^2^/Hz. Electrodes 11 to 13 were analyzed for a frequency spectrum when different lights were shone directly on the exposed brain (Figure 5B–E). As noted in the morphological analysis, the brainwave frequency spectrum for the light-exposed brain is also different from an inside-black-box brain (when the light is off) (C vs. B). There were more alpha (green) and beta (blue) waves present when the brain was exposed to light compared with those present when the light was off. An increment in alpha and beta waves was not only present when the brain was directly exposed to xenon-white light but also when exposed to blue and near-infrared lights (D and E vs. B). Interestingly, theta (yellow) brainwaves were noted to be markedly reduced when the brain was exposed to any light, and delta (red) brainwaves were either reduced (C) or increased (D and E) when exposed to light. In other words, the brain when exposed to either blue or near-infrared light tended to have more delta, alpha, and beta waves. Another interesting difference between blue and near-infrared light direct exposure to the brain was the emergence of gamma waves when near-infrared light was shone on the brain (specifically on the hippocampus), as shown in Figure 6.

## 3. Discussion

The brain is seen as being protected from the light. The scalp, skull, and meningeal layers form barriers that prevent the sun or artificial light from shining on or entering the brain. Therefore, the brain is seen as a black body inside the cranium. The only way for light to enter the brain is through the eyes. The light that enters the eyes will then be processed further at various anatomical levels including the following: optic nerves, chiasm, tracts, radiations, primary visual area Brodmann 17, secondary visual areas Brodmann 18 and 19, and other association areas of the brain. Another way light can significantly enter the cranium is when a neurosurgeon performs a craniotomy or decompressive craniectomy (DC or removal of a large area of cranial bone) for a severely injured brain to relieve raised intracranial pressure. Despite the potential benefit of DC, data from studies demonstrated high incidences of neuropsychological impairments in the long-term follow-up of these DC patients [9,10]. The explanations given for these impairments are a reduction in cerebral perfusion, alteration in cerebrospinal fluid (CSF) dynamics, and atmospheric gravity force pressing the microgravity-brain environment [11,12]. Another possible reason is that light can penetrate the scalp relatively easily in DC patients. Exposure to bright sun or artificial light is known to trigger migraine headaches, as reported in the literature [13,14]. On another note, direct light exposure to the brain has also been reported as having beneficial effects. A study by Naeser et al. (2014) showed significant improvements in cognitive performances in patients with mild head injury [15]. Lately, a significant amount of review articles on photobiomodulation (PBM) have similarly pointed out the benefits of neurodegenerative animal models and cellular studies. The hypothetical mechanisms of PBM offered in their articles are related to the extra synthesis of ATP by mitochondria, diffusion of nitric oxide promoting vasodilation, an increase in regional cerebral blood flow in cortical areas, an increase in antioxidants, augmentation of brain glymphatic drainage system, and a decrease in inflammation [16,17,18,19]. With these notes, our clinical findings, which showed significant alteration in brainwave power and frequency when the brain was exposed directly to various lights concur with the aforementioned positive and negative effects of light on the brain. The right dose of light intensity (luminosity or fluence), type of light, duration, approach or method for light delivery, and area of the brain receiving light are some possible factors that determine the effectiveness and complication of the therapy. Since there are no concrete explanations that can be offered to explain the mechanisms of action for PBM, in this article, we discuss this in accordance with the physics perspective on possible underlying mechanisms for the ‘fast changes’ in brainwaves when four consecutive light conditions were performed during awake brain surgery.

### 3.1. Physics Perspective on Direct Light Energy onto the Brain

Based on our earlier discussion, a significant amount of light energy enters the brain via only the eyes. The black box analogy of the cranium prevents the brain from receiving a significant amount of light. Attenuation of light energy occurs when it passes through layers of the cranium. Therefore, the light resistance property of the cranium may offer a protective mechanism ensuring optimal brain function. Light is a type of electromagnetic energy that can generate heat energy and exists in duality; it can either be regarded as photons (particles of light) or waves [20]. Certainly, heat generation in the brain is related to its metabolism and information processing [21], and with additional exposure to extracranial heat energy (light), brain temperature can be altered. Hence, intricate brain temperature regulation is seen as vital for processing information. The temperature regulation of the brain is a dynamic process and is thought to follow the thermodynamics principles of energy conservation and flow of energy. Energy conservation is in fact the first law of thermodynamics, which states that energy cannot be created or destroyed, it can only change forms or be transferred from one object to another (quantity of energy). It is important to note here that none of the energy-transferring processes are efficient; instead, in each process, some of the transferred energy turns into heat. Inside the cranium, heat energy or temperature could possibly be regulated by heat conduction (via blood flow and CSF), convection (via air sinuses and CSF), evaporation (CSF and air sinuses), and radiation (cranial black box). Pertaining to the flow of energy, the second law of thermodynamics (quality or directionality of energy) states that the entropy of any system always increases. Entropy is defined as a measure of thermal energy per unit temperature that is unavailable for performing useful work, or entropy is a measurement of disorder or energy spread. A low-entropy system has a concentrated energy state, whilst a high-entropy system has a spread-out energy state. It seems fitting with the research findings that the deep brain nuclei and brainstem have warmer temperatures than the cortices [22,23,24]. It tells us the directionality or gradient of energy flow from a low-entropy area such as the brainstem (concentrated energy) to a high-entropy area such as the cortex (spread-out energy). In summary, our arguments cover two salient points as follows: the cranium, as a black box, prevents direct light exposure, and brain thermal homeostasis with thermodynamic principles that regulate brain temperature intricately make physics an aspect to consider deeply when discussing brain functions.

**Figure 7:**
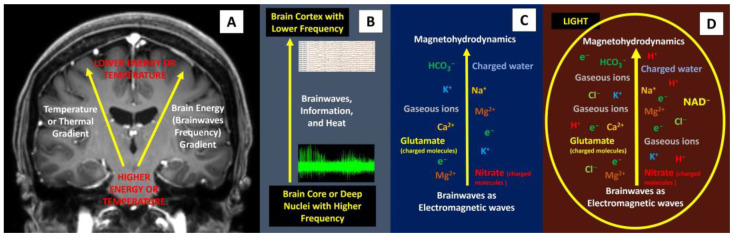
Brainwaves, thermal gradient, and the possible presence of plasma-like brain energy. (**A**,**B**) The presence of a temperature gradient is related closely to brainwaves and information processing inside the brain. (**C**) A combination of brainwaves with extracellular plasma soup (charged ions, charged molecules, charged solvent, gaseous ions, and electrons) may form magnetohydrodynamics force, which has the following peculiar features: rapid, magnetic reconnection and magneto-acoustic waves that help to spread fast and wider brain energy or information. (**D**) Directly shining light on the brain may form more plasma and hence, rapid changes in brainwaves.

Pertaining to ‘rapid brainwave changes’ when different light was shone on the brain, the physics perspective on this matter is likely related to the production of brain plasma. The term ‘plasma’ was first proposed by the American Nobel laureate chemist and physicist Irving Langmuir, who viewed it as similar to blood plasma. Here, ‘plasma’ refers to the electrified fluid that carries electrons and charged ions (compared to how blood plasma carries the corpuscles or cells). In physics, plasma, by definition, must contain an assembly of mobile charge carriers of a sufficient density. If the charged density is not sufficiently high, their interaction with the electromagnetic field becomes important, and the charged component can also be considered plasma [25]. In principle, plasma is commonly divided into ultra-hot (a thermonuclear reaction plasma such as in the sun), hot or thermal plasma, cold plasma (non-thermal or non-equilibrium plasma that can be generated at room temperature), and ultra-cold plasma. The term for hot or cold plasma is sometimes referred to as being hot if it is nearly fully ionized or cold if only a small fraction of it is ionized. In this context, the presence of light inside the cranial black box may have produced or enhanced brain plasma energy, which caused a fast alteration in brainwaves. To comprehend this notion, let us have a look at the brain extracellular compartment (ECC). As we are aware, the brain interstitial or ECC has charged ions (such as Na^+^, Cl^−^, H^+^, Ca^2+^, K^+^, Mg^2+^), charged molecules or polyatomic ions (HCO_3_^−^, hyaluronic acid, NAD^+^, acetate, nitrate, nitrite, sulfate, phosphate, aspartate, glutamate, lysine, arginine, etc.), polar solvents (charged water), gaseous ions (the abundant presence of CO_2_ is easily converted to gaseous ions when electricity or electron movement is applied to them), and electrons (electricity) [26,27,28,29,30]. The interaction between this brain plasma soup and brainwaves (electricity or electron or electromagnetic force) and charged water may result in the formation of magnetohydrodynamics (MHD) or magnetofluid (superfluid) [31,32]. In short, in a slice of alive brain tissue with thousands of neurons, glial cells, and interstitial spaces with charged plasma soup, the possible presence of large amounts of toroidal electromagnetic waves and poloidal plasma waves may form a more diffuse and widespread rapid energy flow (MHD and perpendicular magnetoacoustics waves) from the core to the periphery of the brain (Figure 7). Therefore, the first reason to support this perspective (the brain also has plasma energy) is a black box feature of the cranium, which blocks light from directly reaching the brain and produces additional plasma energy. This idea is supported by a previously mentioned study by Maccaferri et al., which demonstrated nanoplasmon formation when light interacts with electrons (electricity) [7]. The second reason is related to the presence of cortical surface electrons. In 2017, Kiyotaka et al. performed a seven-Tesla MRI study of brain cortical surfaces. Their unexpected finding was an electron-rich layer at glia limitans externa adjacent to CSF cortical cisternal space. Their explanation for the presence of surface electrons adjacent to CSF space was that astrocyte endfeet containing abundant aquaporin-4 (AQP-4) induce glia limitans externa polarization and hence electrons [33]. According to our physics perspective, an interaction between poloidal plasma waves at the cortical surface and water (CSF) may also form abundant electrons, as confirmed by a physics experiment in which electrons were formed when plasma interacted with water [31]. In addition, some recent experiments have also shown the possible presence of plasma-like energy inside the brain, which supports our brain plasma hypothesis and provides an explanation for the rapid changes in brainwaves when light is shone directly on the brain. In 2022, Yidi Zhang et al. noted two types of waves in the brain including front waves of chemical reactions and traveling waves of neural activity [34]. In 2009 and 2021, Andersen et al. and Drukarch et al. highlighted the thermodynamics concept for action potential propagation [35,36]. Finally, could the rapid changes in brainwaves when light is shone on the brain be due to the fundamental brain itself? Indeed, the anatomical brain can also be viewed as waves of energy (i.e., the duality of a particle as either a particle or waves), as highlighted by us in a new perspective for comprehending brain function, where the anatomical brain is viewed as a package of energy or a quantum brain, rather than particles or an anatomical object (Newtonian brain) [37,38,39].

### 3.2. Photobiomodulation (PBM) and Medical Application

Based on our intraoperative findings on the human brain and other clinical–nonclinical findings that showed a positive light effect on neurological diseases, further research in this field is highly encouraged. This could lead to a breakthrough in clinical neurosciences whereby light could play a role in the treatment of various neurological diseases. Matters that need to be considered with regard to PBM therapy include the following: (1) the area of the brain receiving light therapy—focus on deep nuclei/small areas or more diffuse on a large area of the brain such as the prefrontal or orbitofrontal lobe. (2) The type of light and its wavelengths—such as visible light, ultraviolet light, or infrared light. Concerning infrared light, it was used in depression and was shown to have a promising effect [40]. This clinical finding concurs with our finding that near-infrared light does have higher energy power and gamma waves than others. Thus, (near-) infrared light might possibly be used in therapy for depression, blue light for psychosis, and a combination of blue (intensive care stage where electrical seizures may still be present) and infrared (later, in the ward stage when brain energy is low) light for patients with severe head injuries. (3) The light source—coherent or non-coherent light. (4) Approaches for light delivery—transcranial, directly on the brain, or intranasal. (5) The fluence or energy density, which is calculated by multiplying irradiance (W/cm^2^) by time (sec) and is defined as the amount of energy per unit of area (J/cm^2^). Transcranial PBM studies in humans have fluences ranging from 10 to 30 J/cm^2^ [16,41]. (6) The operation mode—continuous wave (CW) or pulsed wave (PW) mode. PW mode has demonstrated significant benefits and better biostimulatory effects than CW mode [42]. Finally, (7) treatment duration and repetition.

## 4. Strengths, Limitations, and Future Directions

Our manuscript’s strength is that ‘human brainwave data’ analysis was performed at specific times when the brain was directly exposed to different types of light available in a standard neurosurgical microscope that was used for optimal surgical viewing and focusing on the area of interest. The obtained data are gold standard brainwave data, which were acquired directly from the brain surface (electrocorticography or ECoG) under the awake state. The findings may also provide additional scientific data concerning brain photobiomodulation, invigorate scientific discussions on brain thermodynamics, and stimulate discussions on the cranium as a black box that houses the brain as a black body and plasma-like brain energy. On the contrary, this manuscript also has some limitations. It is ‘a case report’ depicting results from a single subject in the absence of experimental control and proper statistical analysis. Other limitations include the use of multiple or various types of light and the lack of an analysis of the complications or harmful effects of the light.

## 5. Conclusions

Light has limited access to the brain. The eyes are the only pathway for extracranial light to enter the brain and thus, the intracranial space is regarded as a black box. Therefore, the brain is protected from receiving light directly and can be regarded as a black body that is in thermodynamic equilibrium with its environment. Nonetheless, based on our and other scientific findings, shining direct light on the brain may form a definitive therapy for some neurological and neuropsychiatric disorders in view of its capability to alter brainwaves.

## Data Availability

The detailed presented data are available upon request from the corresponding author. The data are not publicly available due to our hospital data privacy policy.

## References

[1] Wyse C.A., Selman C., Page M.M., Coogan A.N., Hazlerigg D.G. (2011). Circadian desynchrony and metabolic dysfunction; did light pollution make us fat?. Med. Hypotheses.

[2] Yu B., Wang C.Y. (2022). Osteoporosis and periodontal diseases—An update on their association and mechanistic links. Periodontology.

[3] Schulz P., Steimer T. (2009). Neurobiology of circadian systems. CNS Drugs.

[4] Walker W.H., Walton J.C., DeVries A.C., Nelson R.J. (2020). Circadian rhythm disruption and mental health. Transl. Psychiatry.

[5] Wirz-Justice A., Skene D.J., Münch M. (2021). The relevance of daylight for humans. Biochem. Pharmacol..

[6] Cakir M., Colak A., Calikoglu C., Taspinar N., Sagsoz M.E., Kadioglu H.H., Hacimuftuoglu A., Seven S. (2016). Once the Light Touch to the Brain: Cytotoxic Effects of Low-Dose Gamma-Ray, Laser Light, and Visible Light on Rat Neuronal Cell Culture. Eurasian J. Med..

[7] Maccaferri N., Zubritskaya I., Razdolski I., Chioar I.-A., Belotelov V., Kapaklis V., Oppeneer P.M., Dmitriev A. (2020). Nanoscale magnetophotonics. J. Appl. Phys..

[8] Hassan U., Amer F., Hussain M., Mushtaq S., Loya A., Abu Bakar M. (2023). Gemistocytic Differentiation in Isocitrate Dehydrogenase Mutant Astrocytomas: A Histopathological and Survival Analysis. Cureus.

[9] Ballestero M.F.M., Furlanetti L.L., Augusto L.P., Chaves P.H.C., Santos M.V., de Oliveira R.S. (2019). Decompressive craniectomy for severe traumatic brain injury in children: Analysis of long-term neuropsychological impairment and review of the literature. Child’s Nerv. Syst..

[10] Yang X.F., Wen L., Shen F., Li G., Lou R., Liu W.G., Zhan R.Y. (2008). Surgical complications secondary to decompressive craniectomy in patients with a head injury: A series of 108 consecutive cases. Acta Neurochir..

[11] Woo P.Y.M., Mak C.H.K., Mak H.K.F., Tsang A.C.O. (2020). Neurocognitive recovery and global cerebral perfusion improvement after cranioplasty in chronic sinking skin flap syndrome of 18 years: Case report using arterial spin labelling magnetic resonance perfusion imaging. J. Clin. Neurosci..

[12] Idris Z., Mustapha M., Abdullah J.M. (2014). Microgravity environment and compensatory: Decompensatory phases for intracranial hypertension form new perspectives to explain mechanism underlying communicating hydrocephalus and its related disorders. Asian J. Neurosurg..

[13] Tekatas A., Mungen B. (2013). Migraine headache triggered specifically by sunlight: Report of 16 cases. Eur. Neurol..

[14] Lema A.K., Anbesu E.W. (2022). Computer vision syndrome and its determinants: A systematic review and meta-analysis. SAGE Open Med..

[15] Naeser M.A., Zafonte R., Krengel M.H., Martin P.I., Frazier J., Hamblin M.R., Knight J.A., Meehan W.P., Baker E.H. (2014). Significant improvements in cognitive performance post-transcranial, red/near-infrared light-emitting diode treatments in chronic, mild traumatic brain injury: Open-protocol study. J. Neurotrauma.

[16] Salehpour F., Mahmoudi J., Kamari F., Sadigh-Eteghad S., Rasta S.H., Hamblin M.R. (2018). Brain Photobiomodulation Therapy: A Narrative Review. Mol. Neurobiol..

[17] Salehpour F., Gholipour-Khalili S., Farajdokht F., Kamari F., Walski T., Hamblin M.R., DiDuro J.O., Cassano P. (2020). Therapeutic potential of intranasal photobiomodulation therapy for neurological and neuropsychiatric disorders: A narrative review. Rev. Neurosci..

[18] Abijo A., Lee C.Y., Huang C.Y., Ho P.C., Tsai K.J. (2023). The Beneficial Role of Photobiomodulation in Neurodegenerative Diseases. Biomedicines.

[19] Semyachkina-Glushkovskaya O., Abdurashitov A., Klimova M., Dubrovsky A., Shirokov A., Fomin A., Terskov A., Agranovich I., Mamedova A., Khorovodov A. (2020). Photostimulation of cerebral and peripheral lymphatic functions. Transl. Biophotonics.

[20] Rab A.S., Polino E., Man Z.X., Ba An N., Xia Y.J., Spagnolo N., Franco R.L., Sciarrino F. (2017). Entanglement of photons in their dual wave-particle nature. Nat. Commun..

[21] Collell G., Fauquet J. (2015). Brain activity and cognition: A connection from thermodynamics and information theory. Front. Psychol..

[22] Hayward J.N., Baker M.A. (1969). A comparative study of the role of the cerebral arterial blood in the regulation of brain temperature in five mammals. Brain Res..

[23] Wang H., Wang B., Normoyle K.P., Jackson K., Spitler K., Sharrock M.F., Miller C.M., Best C., Llano D., Du R. (2014). Brain temperature, and its fundamental properties: A review for clinical neuroscientists. Front. Neurosci..

[24] Idris Z., Yee A.S., Wan Hassan W.M.N., Hassan M.H., Ab Mukmin L., Mohamed Zain K.A., Manaf A.A., Balandong R.P., Tang T.B. (2023). Clinical outcomes and thermodynamics aspect of direct brain cooling in severe head injury. Surg. Neurol. Int..

[25] Zon Józef R. (2005). Physical Plasma Switchability in the Brain. Electromagn. Biol. Med..

[26] Nakada T. (2009). Neuroscience of water molecules: A salute to Professor Linus Carl Pauling. Cytotechnology.

[27] Nicholson C., Hrabětová S. (2017). Brain Extracellular Space: The Final Frontier of Neuroscience. Biophys. J..

[28] Lei Y., Han H., Yuan F., Javeed A., Zhao Y. (2017). The brain interstitial system: Anatomy, modeling, in vivo measurement, and applications. Prog. Neurobiol..

[29] Purushotham S.S., Buskila Y. (2023). Astrocytic modulation of neuronal signalling. Front. Netw. Physiol..

[30] James E., Jake B., Frederik L., Vindy T., Ian H., Emily H., Collingwood J.F., Telling N.D. (2023). Illuminating the brain: Revealing brain biochemistry with synchrotron X-ray spectromicroscopy. J. Electron Spectrosc. Relat. Phenom..

[31] Bruggeman P.J., Kushner M.J., Locke B.R., Gardeniers J.G.E., Graham W.G., Graves D.B., Hofman-Caris R.C.H.M., Maric D., Reid J.P., Ceriani E. (2016). Plasma–liquid interactions: A review and roadmap. Plasma Sources Sci. Technol..

[32] Peng Y., Alsagri A.S., Afrand M., Moradi R. (2019). A numerical simulation for magnetohydrodynamic nanofluid flow and heat transfer in rotating horizontal annulus with thermal radiation. RSC Adv..

[33] Suzuki K., Yamada K., Nakada K., Suzuki Y., Watanabe M., Kwee I.L., Nakada T. (2017). MRI characteristics of the glia limitans externa: A 7T study. Magn. Reson. Imaging.

[34] Zhang Y., Guo S., Sun M., Mariniello L., Tozzi A., Zhao X. (2022). Front Waves of Chemical Reactions and Travelling Waves of Neural Activity. J. NeuroPhilosophy.

[35] Andersen S.S., Jackson A.D., Heimburg T. (2009). Towards a thermodynamic theory of nerve pulse propagation. Prog. Neurobiol..

[36] Drukarch B., Wilhelmus M.M.M., Shrivastava S. (2021). The thermodynamic theory of action potential propagation: A sound basis for unification of the physics of nerve impulses. Rev. Neurosci..

[37] Idris Z., Zakaria Z., Yee A.S., Fitzrol D.N., Ghani A.R.I., Abdullah J.M., Hassan W.M.N.W., Hassan M.H., Manaf A.A., Heng R.O.C. (2021). Quantum and Electromagnetic Fields in Our Universe and Brain: A New Perspective to Comprehend Brain Function. Brain Sci..

[38] Idris Z. (2020). Quantum Physics Perspective on Electromagnetic and Quantum Fields Inside the Brain. Malays. J. Med. Sci. MJMS.

[39] Idris Z., Yee A.S., Wan Hassan W.M.N., Hassan M.H., Mohd Zain K.A., Abdul Manaf A. (2022). A Clinical Test for a Newly Developed Direct Brain Cooling System for the Injured Brain and Pattern of Cortical Brainwaves in Cooling, Noncooling, and Dead Brain. Ther. Hypothermia Temp. Manag..

[40] Schiffer F., Johnston A.L., Ravichandran C., Polcari A., Teicher M.H., Webb R.H., Hamblin M.R. (2009). Psychological benefits 2 and 4 weeks after a single treatment with near infrared light to the forehead: A pilot study of 10 patients with major depression and anxiety. Behav. Brain Funct. BBF.

[41] Hamblin M.R. (2016). Shining light on the head: Photobiomodulation for brain disorders. BBA Clin..

[42] Peoples C., Spana S., Ashkan K., Benabid A.L., Stone J., Baker G.E., Mitrofanis J. (2012). Photobiomodulation enhances nigral dopaminergic cell survival in a chronic MPTP mouse model of Parkinson’s disease. Park. Relat. Disord..

